# Associations Between Patient-Reported Outcomes and Dual-Task Jump Performance After ACL Reconstruction: Analyses by Sex

**DOI:** 10.1177/19417381251389835

**Published:** 2025-11-18

**Authors:** Alva Lövgren, Andrew Strong, Jonas L. Markström

**Affiliations:** † Department of Community Medicine and Rehabilitation, Unit of Physiotherapy, Umeå University, Umeå, Sweden

**Keywords:** anterior cruciate ligament, dual-tasking, knee injury, patient-reported outcome measures, rehabilitation

## Abstract

**Background:**

Subjective patient-reported outcome measures (PROMs) and physical performance tests are important tools for assessing rehabilitation after anterior cruciate ligament reconstruction (ACLR). However, the associations and interrelationships between PROMs and dual-task jump performance, and sex-specific differences among patients with ACLR remain unclear.

**Hypothesis:**

Associations exist between PROMs and dual-task jump performance, and do not differ between men and women with ACLR.

**Study Design:**

Controlled laboratory study.

**Level of Evidence:**

Level 3.

**Methods:**

A total of 44 sports-active people with ACLR (50% men; mean [SD] 25.4 [16.0] months postsurgery) completed the International Knee Documentation Committee Subjective Knee Form (IKDC-SKF), the Anterior Cruciate Ligament—Return to Sport after Injury (ACL-RSI) scale, and a dual-task drop vertical jump test. The test incorporated cognitive challenges targeting short-term memory, attention, fast decision-making, and inhibitory control. Dual-task performance was quantified as the percentage of correctly completed memory and motor trials. Associations were analyzed using partial correlations, controlling for time post-ACLR.

**Results:**

Across all participants, no significant correlations were observed between dual-task performance and ACL-RSI or IKDC-SKF scores (*r* = 0.02-0.14; *P* = 0.37-0.92). Women demonstrated positive correlations (*r* = 0.39-0.40; *P* = 0.07-0.08) with both PROMs, suggesting a potential association, although shallow linear slopes, whereas men showed negative, nonsignificant correlations (*r* = −0.10 to −0.36; *P* = 0.11-0.65). A high and significant correlation was found between ACL-RSI and IKDC-SKF scores among women (*r* = 0.76; *P* < 0.01) but not men (*r* = 0.36; *P* = 0.11).

**Conclusion:**

After ACLR, ACL-RSI and IKDC-SKF scores were not significantly associated with dual-task jump performance. However, women showed greater consistency between the 2 PROMs than men.

**Clinical relevance:**

The ACL-RSI, IKDC-SKF, and dual-task jump performance capture distinct aspects of recovery, potentially reflecting different biopsychosocial constructs, highlighting the importance of considering sex-specific factors in post-ACLR rehabilitation and assessment.

Anterior cruciate ligament (ACL) injury is the most common season- and career-ending injury for young sportspeople,^2,13^ and injury rates continue to increase, particularly among female adolescents.^4^ Psychological factors, such as self-rated knee function impairment and fear of reinjury, are cited frequently as primary reasons for athletes failing to return to sport (RTS).^3,25^ To assess these factors, clinicians and researchers often use patient-reported outcome measures (PROMs) such as the International Knee Documentation Committee Subjective Knee Form (IKDC-SKF) to assess knee function,^10^ and the Anterior Cruciate Ligament-Return to Sport after Injury (ACL-RSI) scale to assess psychological readiness.^23^

Previous research highlights the importance of PROMs in rehabilitation from ACL reconstruction (ACLR): a systematic review and meta-analysis of >3700 patients found that those who successfully RTS after ACLR reported higher ACL-RSI scale and IKDC-SKF scores than those who did not.^24^ Higher IKDC-SKF scores have also been associated with better quadriceps strength recovery post-ACLR,^11^ while higher ACL-RSI scores relate to better hop performance.^16^ Despite the PROMs distinct focuses, these scales are correlated only moderately (*r* = 0.55-0.60) among patients with ACLR,^7,16,17^ suggesting overlapping constructs. Notably, women tend to report lower scores on both scales compared with men,^14,19^ accompanied by reduced RTS rates,^14^ highlighting the need to explore sex-specific patterns in ACL rehabilitation and whether associations between subjective ratings and physical performance differ by sex.

Objective screening tests, such as strength and jump assessments, are used commonly to evaluate physical performance before recommending RTS but have been criticized for not replicating the unpredictable and dynamic nature of sports.^8,18^ Integrating cognitive tasks with motor tasks may enhance ecological validity and sensitivity.^8,18^ For example, adding secondary cognitive tasks to physical tests has revealed altered movement mechanics linked to higher ACL injury risk,^12,21^ and biomechanical outcomes of incomplete ACL rehabilitation.^20^ Athletes with ACLR also perform worse on cognitive-motor dual-task jump testing than healthy controls, highlighting the need to assess dual-task ability.^20^ Examining how ACL-RSI and IKDC-SKF scores relate to dual-task performance, both overall and by sex, may enhance clinical interpretation of these PROMs and support safer, more effective RTS decisions.

This study aimed to investigate the associations between the ACL-RSI and IKDC-SKF scores and dual-task drop vertical jump (DVJ) performance, as well as the relationship between the 2 scales themselves, and to contrast these associations between men and women with a history of ACLR. We hypothesized that PROMs would be associated with dual-task jump performance and would not differ between male and female patients with ACLR.

## Methods

### Study Design and Setting

This cross-sectional study was performed in a research laboratory and was approved by the National Ethical Review Authority (Dnr. 2023-00342-01). Participants provided written informed consent before participating, and the study was conducted per the ethical principles stated in the Declaration of Helsinki.

### Participants

The study involved 44 people (22 men) with a history of ACLR, of whom 4 people had ruptured the ACL on both legs (Table 1). The mean time between ACLR and testing (the latest for those with reinjury) was 25.4 (SD 16.0, range 8 to 59) months. Participants were recruited using a register from the orthopaedic clinic of the regional hospital and local outreach. Inclusion criteria were: 15 to 36 years of age (typical age for adult-level competition), a Tegner activity scale rating ≥6 out of 10,^22^ current weekly participation in sports involving rapid and unpredictable changes (eg, ball sports; high ACL injury rate), confident of performing maximal hop and strength tests (based on interview), hamstring graft (standard national practice), no concomitant injuries, no severe ankle sprain within 6 months, and no other conditions affecting hopping ability.

**Table 1.** Participant (n = 44, 50% men) demographics at time of testing

Outcome	
Age, years	26.1 (5.6)
Time since ACLR, months	25.4 (16.0)
Body mass, kg	72.8 (11.2)
Body height, m	1.72 (0.10)
Body mass index, kg/m^2^	24.6 (2.6)
Tegner rating, score	7.6 (1.3)
IKDC activity level 1, n (%)*^a^*	38/44 (86.4%)
IKDC activity level 2, n (%)*^a^*	6/44 (13.6%)
ACL-RSI, %	61.4 (18.5)
IKDC-SKF, %	83.1 (9.8)

Values are mean (SD) unless stated otherwise. ACL, anterior cruciate ligament; ACLR, ACL reconstruction; ACL-RSI, ACL - Return to Sport after Injury; IKDC-SKF, International Knee Documentation Committee Subjective Knee Form.

a From Hefti et al^9^, defining IKDC activity levels 1-4.

### Study Procedures

Participants first completed the ACL-RSI and IKDC-SKF, both of which are reliable and valid for people with ACL injury.^6,10,23^ The ACL-RSI is a 12-item questionnaire that assesses psychological readiness for RTS, with a lower total score indicating less readiness. The IKDC-SKF is a 10-item questionnaire evaluating self-rated overall knee function, with a lower total score reflecting poorer function.

Wearing their own sports clothing (shoes, short tights, and sports bra if female), participants then completed a standardized warm-up: 2 sets of 6 body-weighted squats, 3 lunges per leg, and 3 squat jumps, followed by 2 to 3 practice trials without and with secondary cognitive tasks. They then performed the dual-task DVJ test, which included cognitive tasks targeting attention, fast decision-making, inhibitory control, and short-term memory, as previously detailed.^20^ Briefly, participants stood on a 35-cm-high wooden platform and memorized the positions of letters A-F displayed on a TV screen placed about 5 meters ahead. After 5 seconds, the letters disappeared, and the participants dropped down and ~50 cm forward. Upon leaving the platform, a black or red arrow containing one of the letters appeared on the screen for 1 second (elements of attention and fast decision-making). A black upward arrow signaled to land and immediately jump as high as possible, while a black downward arrow signaled to land only. Red arrows indicated the opposite action of the corresponding black arrows (element of inhibitory control). Immediately after the motor task, participants recalled the pre-drop position of the letter shown (element of short-term memory). The order of the letters and the letter inside the arrow were randomized between trials. Instructions were given during warm-up and before testing: “Land softly and as quietly as possible, and if jumping after the landing, push the ground away as hard as you can to jump as high as possible.” Each arrow type (up/down, black/red) was presented 3 times in randomized, counterbalanced order, totaling 12 trials.

The outcome variable was the percentage of correct memory and motor elements from all 12 trials (maximum score = 24). The test leader noted correct letter-position recalls and motor actions, which had to be performed smoothly in 1 motion.

Arrows and letters during the test were triggered by a custom-built system incorporating a laser-ranging time-of-flight sensor (ST VL53L1X) mounted on the box and aimed at the participants’ heels. The sensor detected movement events, which were processed by a microcontroller (Arduino UNO) and communicated via a serial interface to custom software running on a computer connected to the TV. The same test leader instructed all participants.

### Statistical Analysis

Partial correlations (Pearson’s *r*) were used to determine the linear associations between dual-task performance and the ACL-RSI and IKDC-SKF scores and between the 2 PROMs while controlling for time post-ACLR for men and women together as well as separately. The strength of the correlations was interpreted as follows: 0.00-0.30 negligible, 0.31-0.50 low, 0.51-0.70 moderate, 0.71-0.90 high and 0.91-1.00 very high.^1^ The Statistical Package for the Social Sciences (Version 23, IBM SPSS Statistics) was used for all analyses with a 5% significance level set a priori.

## Results

**Figure 1.**
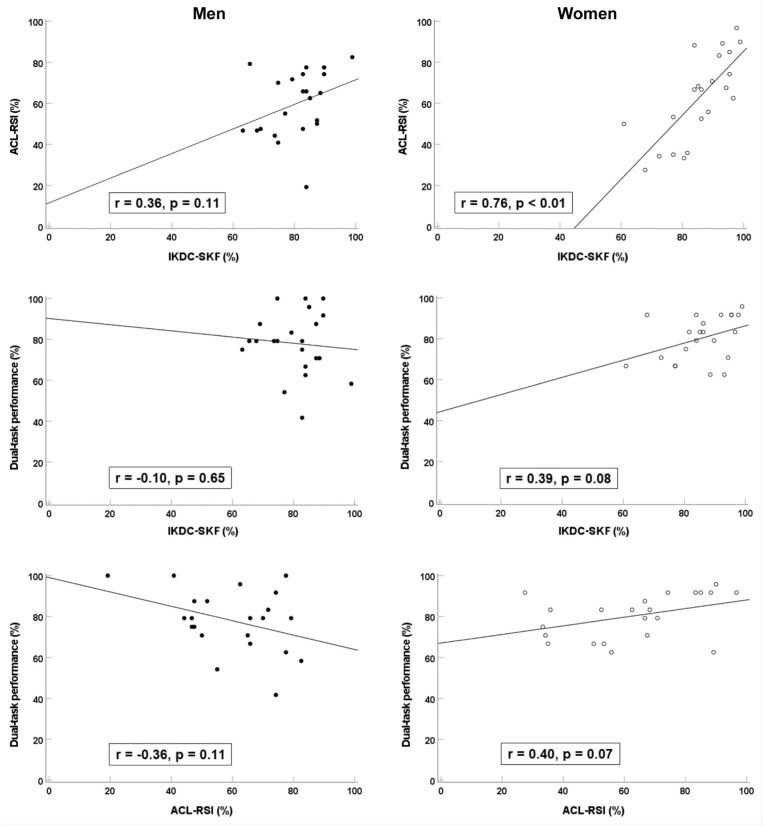
Scatterplots with results from partial correlations (controlling for time post-ACLR) illustrating linear trends between the PROMs ACL-RSI and IKDC-SKF, and cognitive-motor dual-task jump performance, shown separately for men and women with ACLR. ACL, anterior cruciate ligament; ACLR, ACL reconstruction; ACL-RSI, ACL-Return to Sport after Injury; IKDC-SKF, International Knee Documentation Committee Subjective Knee Form; PROM, patient-reported outcome measure.

Including all participants, no significant correlation was found between dual-task performance and ACL-RSI (*r* = 0.02; *P* = 0.92) or IKDC-SKF (*r* = 0.14; *P* = 0.37). When analyses were performed separately by sex, female participants showed weak positive correlations between dual-task performance and both ACL-RSI and IKDC-SKF, whereas male participants demonstrated weak negative correlations between dual-task performance and ACL-RSI and IKDC-SKF, none of which reached statistical significance. A strong, statistically significant positive correlation was observed between ACL-RSI and IKDC-SKF scores in women. In contrast, a weak, nonsignificant positive correlation was found in men (Figure 1).

## Discussion

We observed no statistically significant correlations between the PROMs (ACL-RSI and IKDC-SKF) and dual-task performance during DVJs among athletes with a history of ACLR, either with women and men combined or separately. Female participants showed positive correlations between each PROM and dual-task performance, suggesting a potential association, whereas male participants exhibited no meaningful associations between either PROM and dual-task performance. Notably, women also had a strong, significant correlation between the 2 PROMs, which was absent among men. To our knowledge, this is the first study to investigate the associations between the ACL-RSI and IKDC-SKF questionnaires and dual-task performance in this population.

Our findings of weak correlations between these PROMs and dual-task performance suggest they capture distinct constructs. These results align with previous research showing negligible-to-weak correlations between PROMs and physical performance metrics such as single-leg hop performances and leg strength tests in individual patients (men and women combined) with ACLR.^1,15,16^ For example, Meierbachtol et al^15^ reported modest positive correlations (*r* = 0.27-0.28) between ACL-RSI scores and limb symmetry in diverse hop performances on average ~8 months post-ACLR. Al-Gburi et al^1^ found similar associations (*r* = 0.00-0.45) between limb symmetry in various hop tests and multiple PROMs, including ACL-RSI and IKDC-SKF, ~12 months post-ACLR. Likewise, Mercurio et al^16^ observed positive correlations between ACL-RSI and limb symmetry for hop distance (*r* = 0.21) and timed 6-m hop (*r* = 0.28) ~6 months post-ACLR. Our results extend these findings to a more complex dual-task test, reinforcing that PROMs, particularly ACL-RSI and IKDC-SKF, may not fully reflect functional performance.

Although women showed positive correlations between each PROM and dual-task performance, suggesting a trend toward an association, the slopes were low and likely too small to be clinically meaningful (Figure 1). However, the stronger correlation observed between ACL-RSI and IKDC-SKF in women compared with men indicates that PROMs may be more internally consistent in women. Previous studies report moderate correlations (*r* = 0.55-0.60) between ACL-RSI and IKDC-SKF when sexes are combined (sex-specific data is lacking),^7,16,17^ which is consistent with our combined result (*r* = 0.58). These findings were irrespective of time post-ACLR, ranging from group averages of 6 months to several years.^7,16^

Differences between sexes in self-perception may also contribute to the observed difference in PROM alignment. Previous research indicates that men generally report higher self-esteem than women, particularly in Western countries.^5^ Although the translation of this trend to people with ACLR and related PROMs is not fully understood, studies have shown that men report higher ACL-RSI and IKDC-SKF scores,^14,17,19^ but not always for IKDC-SKF.^17^ In our sample, ACL-RSI and IKDC-SKF ratings were comparable between sexes (ACL-RSI: men 59.8 [SD 16.0], women 63.0 [SD 21.0]; IKDC-SKF: men 80.5 [SD 9.0], women 85.6 [SD 10.1]).

### Strengths and Limitations

All participants had undergone ACLR using a hamstring tendon graft, and concomitant injuries were screened via preparticipation interviews. Rehabilitation protocols varied and, as this was an exploratory study, no a priori power analysis was performed. Only athletes who had achieved RTS were included, which may have reduced variability in PROM scores and performance outcomes,^3,25^ potentially underestimating associations in the broader ACLR population. Our sample of recreational and competitive athletes reflected real-world variability in age, sport type, and time since surgery, enhancing generalizability while possibly limiting reliability for subgroup comparisons. Finally, the use of a cognitive-motor dual-task test likely increased the ecological validity of the jump testing.^8,18^

## Conclusion

Outcomes from the PROMs ACL-RSI and IKDC-SKF were not correlated significantly with cognitive-motor dual-task performance among people with a history of ACLR, whether analyzed for men and women combined or separately, suggesting that these measures capture different constructs. Women demonstrated a strong, significant correlation between ACL-RSI and IKDC-SKF, whereas no such correlation was evident for men, indicating greater consistency across PROMs in women. These findings underscore the importance of a comprehensive, holistic approach to ACLR rehabilitation, in which each assessment method contributes uniquely to understanding recovery, and highlights the need for further research to determine whether PROMs should be interpreted separately by sex.
